# Rare variants in *BRCA2* and *CHEK2* are associated with the risk of urinary tract cancers

**DOI:** 10.1038/srep33542

**Published:** 2016-09-16

**Authors:** Yuqiu Ge, Yunyan Wang, Wei Shao, Jing Jin, Mulong Du, Gaoxiang Ma, Haiyan Chu, Meilin Wang, Zhengdong Zhang

**Affiliations:** 1 Department of Environmental Genomics, Jiangsu Key Lab of Cancer Biomarkers, Prevention and Treatment, Collaborative Innovation Center For Cancer Personalized Medicine, Nanjing Medical University, Nanjing, China; 2 Department of Genetic Toxicology, The Key Laboratory of Modern Toxicology of Ministry of Education, School of Public Health, Nanjing Medical University, Nanjing, China; 3 Department of Urology, Huai-An First People’s Hospital Affiliated to Nanjing Medical University, Huai-An, China

## Abstract

Previous studies have shown that two rare variants, rs11571833 in *BRCA2* and rs17879961 in *CHEK2* were associated with lung cancer. However, the associations between these two variants and urinary tract cancers risk remain largely unexplored. We applied imputation of three genome-wide association studies published in the database of Genotypes and Phenotypes (dbGaP). Unconditional logistic regression analysis and meta-analysis were performed to assess the association between these two variants and the risk of urinary tract cancers. Our results showed that rs11571833[T] had an effect on urinary tract cancers predisposition (OR_meta_ = 1.45, *P*_meta_ = 0.013), especially associated with increased the risk of bladder cancer (OR_meta_ = 1.60, *P*_meta_ = 0.010). Moreover, rs17879961[C] had a protective effect on the urinary tract cancers (OR_meta_ = 0.67, *P*_meta_ = 1.0 × 10^−3^) and was mostly associated with a lower incidence of renal cell carcinoma (OR_meta_ = 0.51, *P*_meta_ = 2.0 × 10^−3^). Together, our study indicates that *BRCA2* and *CHEK2* play an important role in the genetic susceptibility to urinary tract cancers.

According to statistics, approximately 429,800 newly diagnosed cases of bladder cancer occurred and caused 165,100 deaths in 2012 worldwide^1^. For prostate cancer, about 1.1 million new cases accounts for the second frequently diagnosed cancer^1^. Besides, renal cell carcinoma is the seventh most common cancer and arises about 209,000 new cases and 102,000 deaths per year^2^. Urinary tract cancers have a serious harm to human health^3^. Smoking is the most well-known risk factor for bladder cancer and renal cell carcinoma, which could increase the risk to be more than 2-fold compared with nonsmokers. There are other environmental risk factors which have been reported to increase risk of urinary tract cancers, such as occupational exposure and obesity^1^,^4^. Epidemiology studies have indicated that except for environmental risk factors, genetic susceptibility and their interaction with environmental factors play a key role in the pathogenesis of cancers^5^,^6^,^7^.

Genome-wide association study (GWAS) and its corresponding meta-analysis have successfully identified several risk loci and regions, which had small or moderate effect to increase the risk of diseases^8^,^9^,^10^. Presently, multiple urinary tract cancers risk–associated SNPs are mostly common and locate in non-coding regions of the genome^11^,^12^,^13^,^14^. However, these risk-associated SNPs contribute little or moderate effect to heritability of urinary tract cancers. The Common Disease-Rare Variant Hypothesis suggests that the additional heritability can be explained by low frequency and rare variants with stronger effect^8^,^15^,^16^.

In recent, an imputation-based genome wide association study had identified two rare variants rs11571833 (c.9976A > T) in *BRCA2* (OR = 2.47, *P* = 4.74 × 10^−20^) and rs17879961 (c.470T > C) in *CHEK2* (OR = 0.38, *P* = 1.27 × 10^−13^), which have large effect on lung cancer in populations of European ancestry^17^. *BRCA2* and *CHEK2*, two important DNA repaired genes, acted a crucial role in response to DNA alterations and involved in maintenance of genome stability^18^,^19^,^20^. Long-term exposure of smoking may result in DNA accumulates damage, which can trigger cell-cycle deregulation and oncogenesis^21^,^22^. Germline rare variation of *BRCA2* and *CHEK2* that changes amino acid may alter their function of DNA damage repair, which may influence the risk of diseases^23^. Previous studies have shown that *BRCA2* and *CHEK2* confer susceptibility to multi-organ cancers, such as breast, lung, prostate and bladder cancer^24^,^25^,^26^,^27^,^28^,^29^. Furthermore, mounting evidence shows chemical carcinogens and reactive oxygen can induce damage to DNA in urothelial cells and polymorphisms in DNA repair genes might modify urothelial carcinoma risk^30^. In addition, the exposure of carcinogens was thought to be similar throughout the urinary tract and the urinary tract cancers are often multifocal^31^. We performed imputation analysis from dbGaP database to explore the association between rs11571833 and rs17879961 polymorphism and the risk of urinary tract cancers in the populations of European descent.

## Results

### The characteristics of GWAS studies for urinary tract cancers

A total of three GWAS studies including 6,064 cases and 8,661 controls were enrolled in our study. The detailed characteristics of GWAS studies are shown in Table 1. In addition, the principal component analysis (PCA) showed there were no abnormal outlier samples in the study population (Supplementary Fig. 1).

### Association analysis for individual GWAS data and joint (pooled) GWAS data

Our logistic regression results are applied in Tables 2 and 3. The imputation info of the two rare variants was mostly more than 0.9, which indicated the accuracy of our imputation result was high. The rs11571833[T] showed significant association with bladder cancer in Illumina 610 array (OR = 1.87, 95%CI = 1.21–2.87, *P* = 4.5 × 10^−3^). Simultaneously, rs17879961[C] was related with decreased the risk of renal cell carcinoma in Illumina 610 array and bladder cancer in Illumina 610 array (OR = 0.50, 95%CI = 0.33–0.77, *P* = 1.5 × 10^−3^ and OR = 0.71, 95%CI = 0.52–0.98, *P* = 0.036, separately).

In consideration of the relative small sample, we attempted to combine the original GWAS data to implement joint analyses. We merged genotype data across each cancer (three for bladder cancer and three for renal cell carcinoma) to increase the sample size. Totally, the sample size is 3591 cases and 4132 controls for bladder cancer, 1322 cases and 3428 controls for renal cell carcinoma, 1151 cases and 1101 controls for prostate cancer. Our results indicated that rs11571833[T] was significantly associated with increased the risk of bladder cancer (OR_combined_ = 1.70, 95%CI = 1.19–2.42 and *P*_combined_ = 3.6 × 10^−3^), but showed non-significant association between renal cell carcinoma and prostate cancer (Table 2). rs17879961[C] had a correlation with renal cell carcinoma (OR_combined_ = 0.63, 95%CI = 0.44–0.89 and *P*_combined_ = 1.0 × 10^−2^). There were no remarkable associations between rs17879961 and the other two cancers (Table 3). For the total three cancers, the results revealed that rs11571833[T] could increase 47% risk of urinary tract cancers (OR_combined_ = 1.47, 95%CI = 1.12–1.94 and *P*_combined_ = 5.7 × 10^−3^) and rs17879961[C] was associated with a significantly lower risk of urinary tract cancers (OR_combined_ = 0.73, 95%CI = 0.59–0.90 and *P*_combined_ = 3.7 × 10^−3^). In conclusion, rs11571833 may play a risk factor in urinary tract cancers predisposition, especially be associated with the risk of bladder cancer. Meanwhile, rs17879961 is associated with decreased risk of urinary tract cancers and mostly associated with the risk of renal cell carcinoma.

### Meta-analysis across multiple GWAS databases

**Figure 1:**
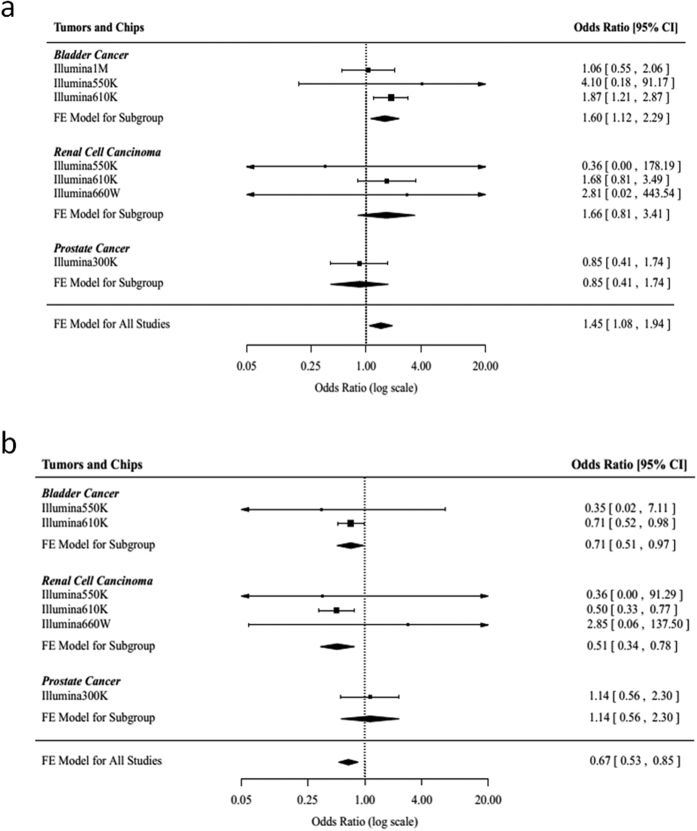
Association between rs11571833 and rs17879961 and urinary tract cancers risk. (**a,b**) Meta-analysis across multiple GWAS databases for each cancer. The result is weighted according to the inverse of the variance of the log of the OR calculated by unconditional logistic regression. Squares boxes indicate the odds ratios and the size of the box is proportional to the weight of the study. Dashed vertical lines represent the null value (OR = 1.0). Horizontal lines represent the 95% confidence intervals. Diamonds indicate the overall summary estimate derived from a fixed-effects (FE) model.

Except for joint analysis, we also applied meta-analysis to assess the association between the two rare variants and urinary tract cancers. For rs11571833, we performed a meta-analysis from seven imputation association results and found that the association between rs11571833[T] and the risk of urinary tract cancers was significant (OR_meta_ = 1.45, 95%CI = 1.08–1.94 and *P*_meta_ = 0.013). In addition, a significant association between rs11571833[T] and bladder cancer was observed in the subgroup meta-analysis (OR_meta_ = 1.60, 95%CI = 1.12–2.29 and *P*_meta_ = 0.010; Table 4 and Fig. 1a). The result showed that rs17879961[C] was associated with decreased the risk of urinary tract cancers (OR_meta_ = 0.67, 95%CI = 0.53–0.85 and *P*_meta_ = 1.0 × 10^−3^) and had a protective effect on renal cell carcinoma (OR_meta_ = 0.51, 95%CI = 0.34–0.78 and *P*_meta_ = 2.0 × 10^−3^; Table 5 and Fig. 1b) by fixed effect model.

## Discussion

Genome stability is critical for preventing tumorigenesis. DNA damages can result in the activation of oncogenes and inactivation of tumor suppressor genes. DNA damage repaired systems involve in maintenance of genome stability and supply a crucial defense function against DNA-damaging agent, such as exposure of cigarette smoking and ultraviolet component of ionizing radiation, sunlight and genotoxic substance^22^,^32^. The main DNA damage repaired pathways include nucleotide-excision repair (NER), base-excision repair (BER), homologous recombination and end joining^33^. Previous studies have highlighted that inherited defects in the DNA repaired pathways may predispose to malignancy^22^.

Recently, an imputation study identified two rare variants rs11571833 in *BRCA2* and rs17879961 in *CHEK2* were associated with lung cancer. *CHEK2* (checkpoint kinase 2 checkpoint homologue) plays an important role in encoding a pluripotent kinase which can induce cell cycle arrest or apoptosis in response to unrepaired DNA damage^34^,^35^. The missense variant rs17879961 (p.Ile157Thr) changes Isoleucine to Threonine at position 157 of the protein and it locates in a region coding for a functionally important FHA domain of *CHEK2* and injures binding of principal substrates. The rs17879961 (*CHEK2* p.Ile157Thr) substitution may alter its ability to bind p53, BRCA1 and Cdc25A proteins^4^,^36^,^37^. *BRCA2* (breast cancer early onset 2) is a widely known anti-oncogene and associated with the risk of breast cancer and ovarian cancer^38^. *BRCA2* also involves in the maintenance of genome stability through interacting with RAD51 recombinase, specifically in the homologous recombination pathway for DNA repair^39^,^40^,^41^. The variant rs11571833 (p.Lys3326X) leads to a stop codon, which results in loss of the final 93 amino acids of the BRCA2 protein. The interaction of RAD51 and BRCA2 plays a crucial role in BRCA2-mediated double strand-break repair. The C-terminus of BRCA2 contains a RAD51 binding domain and small protein sequence incorporating p.Lys3326X (amino acids 3265–3330) is capable of binding RAD51. Besides, an important serine involved in BRCA2-mediated repair process is close to this truncating mutation^41^,^42^,^43^. Above evidence invites that the SNP rs11571833 is functional in DNA damage repair pathway thus alter the genetic susceptibility of cancers. Previous studies have found that rs11571833 was associated with the risk of upper aerodigestive tract cancer (OR = 2.53)^21^. Besides, some studies suggest that rs11571833 have an association with risk of breast cancer^42^. Meanwhile, rs17879961 have been reported to be associated with a significantly lower incidence of lung or upper aerodigestive tract cancer, but increased the risk of thyroid cancer^34^,^44^,^45^. However, the effect of these two rare variants on urinary tract cancers remains largely unexplored.

In this study, we investigated the associations between rare genetic variants of rs11571833 and rs17879961 and the risk of urinary tract cancers (including bladder cancer, prostate cancer and renal cell carcinoma) and found that the rare variant rs11571833 (c.9976A > T) was associated with increased risk of urinary tract cancers, especially associated with bladder cancer. Concurrently, rs17879961 (c.470T > C) played a protective role in the urinary tract cancers carcinogenesis and mostly decreased the risk of renal cell carcinoma. Our meta-analyses result show highly consistency with joint analyses, which strengthen our conclusion that the rs11571833 is associated with increased the risk of urinary tract cancers and rs17879961 reduces urinary tract cancers predisposition. Interestingly, association signals of these two variants in urinary tract cancers (rs11571833, OR = 1.47 ; rs17879961, OR = 0.73) are in the same direction to lung cancer (rs11571833, OR = 2.47; rs17879961, OR = 0.38). It is noteworthy that the rare variants rs17879961 may impair function of CHEK2, but it was associated with decreased the risk of renal cell cancer. A speculation for the possible protective mechanism is that CHEK2 can have two opposite effects on damaged stem cells and it impedes stem cell division until DNA damage has been repaired or actives apoptosis if unrepaired DNA damage happened. Accumulating evidence has shown that in the circumstance of continued DNA damage by long-term exposure of tobacco, the normal stem cell defenses that involve CHEK2 can be attenuated by reducing the CHEK2 activity as a result of rs17879961 (p.Ile157Thr)^17^,^32^,^34^.

There are several limitations in our study. Firstly, the available GWAS data published in dbGaP database provides relatively little samples, which may influence the power to identify the association between the rare variants and diseases. Furthermore, our analysis based on public database is short of relevant demography information applied in stratification analysis. Additionally, our study is an imputation-based analysis from public database and the ORs of two rare variants are relatively modest. Besides, the sample sizes of controls are unevenly matched with cases in some cohorts, which may result in sample bias. Further large well-designed studies in other independent populations and functional studies are needed to validate our findings. Meanwhile, the interaction between environmental exposures and genetic susceptibility should be also considered in the future research^32^.

In conclusion, our imputation analysis results indicated that the rare variant rs11571833 (c.9976A > T) showed an effect on urinary tract cancers predisposition, especially associated with increased the risk of bladder cancer. However, rs17879961 (c.470T > C) may play a protective role in the urinary tract cancers carcinogenesis and notably decrease the risk of renal cell carcinoma. Association signals of these two variants in urinary tract cancers are in the same direction to lung cancer. These results suggest that *BRCA2* and *CHEK2* play an important role in the genetic susceptibility to urinary tract cancers.

## Materials and Methods

### Study population

GWAS data from three cancer studies were available, which were requested from dbGaP. Two genotype datasets were excluded because of low-density genotype panel and low sample size (These two datasets are genotyped by Illumina 240 array and Illumina 317 array separately, and their genome build are both build 35). In the final analysis, we included three bladder cancer studies, three renal cell carcinoma studies and one prostate cancer study, totally including 6,064 cases and 8,661 controls.

### Quality control

First, we applied the LiftOver tool to convert study data from earlier genome builds (NCBI build 36) to NCBI build 37. Few SNPs that failed in the LiftOver were excluded from the imputation. Before performing imputation, we filtered the data to remove low-quality variants and individuals to increase the accuracy of the results. For individuals, (1) we excluded those missing rate per person >5%; (2) excluded samples with ambiguous gender; (3) additional duplicates or probable relatives were excluded according to PI_HAT > 0.5; SNPs filter criterion : (1) completion rate per locus <95%; (2) minor allele frequency <0.05; (3) Hardy-Weinberg Proportion for autosomal SNPs in control population with *P* < 1.0 × 10^−6^; (4) A/T or C/G SNPs with MAF >0.45.

### Imputation analyses

We used SHAPEIT, a highly accurate phasing algorithm, to infer the haplotypes underlying genotype data^46^, then inputting into estimated GWAS haplotypes with IMPUTE2 for imputation. Imputation was conducted separately for each scan by IMPUTE2 Version 2.3.1 and using the 1000 Genomes Project data (version 3, March 2012 release) as reference dataset^47^. We executed imputation across a 5Mb genomic region that included rs11571833 and rs17879961 (chr13: 30472626–35472626 and chr22: 26621087–31621087 respectively). Test of association was performed by SNPTEST Version 2.5 software^48^.

### Statistical analyses

An unconditional logistic regression model was applied to calculate the odds ratio (OR) and 95% confidence interval (CI) for each SNP in an additive model using SNPTEST software. We adjusted for some covariates such as age, gender, study and significant principle components (PCs). The principal component analysis (PCA) was carried out to assess for population stratification of study cohorts. Meta-analysis was conducted by Stata v.10 (Stata College, Taxas, US). Heterogeneity was evaluated by Cochran’Q and *I*^2^ statistics. *I*^2^ values ≥ 75% were considered to be significant heterogeneity. All statistical analyses were two-sided, and a *P* value < 0.05 was considered statistically significant.

## Additional Information

**How to cite this article**: Ge, Y. *et al.* Rare variants in *BRCA2* and *CHEK2* are associated with the risk of urinary tract cancers. *Sci. Rep.* **6**, 33542; doi: 10.1038/srep33542 (2016).

## Supplementary Material

Supplementary Information

## Figures and Tables

**Table 1:** Description of GWASs for urinary tract cancers.

Tumor types	Cases	Controls	Platform	Published GWAS
Bladder cancer	1106	1048	Illumina Human1Mv1_C	Rothman^49^
(phs000346.v1.p1)	38	825	Illumina HumanHap550v3.0	
	2447	2259	Illumina Human610_Quadv1_B	
Renal cell carcinoma	9	845	Illumina HumanHap550v1.1	Purdue^13^
(phs000351.v1.p1)	672	2575	Illumina Human610_Quadv1_B	
	641	8	Illumina Human660W-Quad_v1_A	
Prostate cancer (phs000207.v1.p1)	1151	1101	Illumina HumanHAP300V1.1	Yeager^50^

**Table 2:** Logistic regression analysis of rs11571833 (c.9976A > T) in BRCA2 and three urinary tract cancers in individual GWAS data.

Study	Info^a^	Case/control	Maf^b^ case/control	OR^c^ (95%CI)	*P* value^c^
Bladder cancer_1M	0.929	1106/1048	0.0087/0.0082	1.06 (0.55–2.06)	0.860
Bladder cancer_550	0.888	38/825	0.0132/0.0054	4.10 (0.18–91.17)	0.373
Bladder cancer_610	0.896	2447/2259	0.0127/0.0072	1.87 (1.21–2.87)	4.5E-03
Bladder caner_combined	0.872	3591/4132	0.0114/0.0073	1.70 (1.19–2.42)	3.6E-03
Renal cell carcinoma_550	0.899	9/845	0.0002/0.0060	0.36 (0.00–178.19)	0.746
Renal cell carcinoma_610	0.882	672/2575	0.0107/0.0072	1.68 (0.81–3.49)	0.166
Renal cell carcinoma_660	0.820	641/8	0.0098/0.0000	2.82 (0.02–443.54)	0.688
Renal cell carcinoma_combined	0.865	1322/3428	0.0097/0.0068	1.60 (0.91–2.82)	0.103
Prostate cancer	0.875	1151/1101	0.0071/0.0082	0.85 (0.41–1.74)	0.647
Three cancers_combined	0.874	6064/8661	0.0099/0.0071	1.47 (1.12–1.94)	5.7E-03

^a^Info: A measure of the observed statistical information for the estimate of allele frequency of the SNP using all individuals in the sample that are used for the test at each SNP. This measure has a maximum value of 1 that indicates that perfect information.

^b^Maf: minor allele frequency.

^c^OR, odds ration; CI, confidence interval. *P* was for addictive model.

**Table 3:** Logistic regression of rs17879961 (c.470T > C) in CHEK2 and three urinary tract cancers in individual GWAS data.

Study	Info^a^	Case/control	Maf^b^ case/control	OR^c^ (95%CI)	*P* value^c^
Bladder cancer_1M	0.297	1106/1048	0.0002/0.0000	NA^d^	NA
Bladder cancer_550	0.930	38/825	0.0000/0.0061	0.35 (0.02–7.11)	0.491
Bladder cancer_610	0.973	2447/2259	0.0141/0.0196	0.71 (0.52–0.98)	0.036
Bladder caner_combined	0.966	3591/4132	0.0096/0.0118	0.81 (0.60–1.10)	0.183
Renal cell carcinoma_550	0.917	9/845	0.0000/0.0072	0.36 (0.00–91.29)	0.717
Renal cell carcinoma_610	0.975	672/2575	0.0099/0.0237	0.50 (0.33–0.77)	1.5E-03
Renal cell carcinoma_660	0.955	641/8	0.0170/0.0000	2.85 (0.06–137.50)	0.596
Renal cell carcinoma_combined	0.912	1322/3428	0.0125/0.0199	0.63 (0.44–0.89)	0.010
Prostate cancer	0.972	1151/1101	0.0076/0.0067	1.14 (0.56–2.30)	0.720
Three cancers_combined	0.971	6064/8661	0.0104/0.0142	0.73 (0.59–0.90)	3.7E-03

^a^Info: A measure of the observed statistical information for the estimate of allele frequency of the SNP using all individuals in the sample that are used for the test at each SNP. This measure has a maximum value of 1 that indicates that perfect information.

^b^Maf: minor allele frequency.

^c^OR, odds ration; CI, confidence interval. *P* was for addictive model.

^d^NA, not available.

**Table 4:** Meta-analysis of rs11571833 (c.9976A > T) in BRCA2 and three urinary tract cancers in individual GWAS data.

Study	OR (95%CI)^a^	*P* value	*P*_het_^c^
Bladder cancer_1M	1.06 (0.55–2.06)	0.860	
Bladder cancer_550	4.10 (0.18–91.17)	0.373	
Bladder cancer_610	1.87 (1.21–2.87)	4.5E-03	
Meta-analysis of three bladder cancers studies	1.60 (1.12–2.29)^b^	0.010	0.314
Renal cancer_550	0.36 (0.00–178.19)	0.746	
Renal cancer_610	1.68 (0.81–3.49)	0.166	
Renal cancer_660	2.81 (0.02–443.54)	0.688	
Meta-analysis of three renal cancers studies	1.66 (0.81–3.41)^b^	0.167	0.871
Prostate cancer	0.85 (0.41–1.74)	0.647	
Meta-analysis of all studies	1.45 (1.08–1.94)^b^	0.013	0.521

^a^OR, odds ration; CI, confidence interval. *P* was for addictive model.

^b^OR and 95%CI were estimated from a fixed-effects model.

^c^*P* value of Cochran’s Q-test for the heterogeneity.

**Table 5:** Meta-analysis of rs17879961 (c.470T > C) in CHEK2 and three urinary tract cancers in individual GWAS data.

Study	OR (95%CI)^a^	*P* value	*P*_het_^c^
Bladder cancer_1M	NA^d^	NA	
Bladder cancer_550	0.35 (0.02–7.11)	0.491	
Bladder cancer_610	0.71 (0.52–0.98)	0.036	
Meta-analysis of three bladder cancers studies	0.71 (0.51–0.97)^b^	0.031	0.642
Renal cancer_550	0.36 (0.00–91.29)	0.717	
Renal cancer_610	0.50 (0.33–0.77)	1.5E-03	
Renal cancer_660	2.85 (0.06–137.50)	0.596	
Meta-analysis of three renal cancers studies	0.51 (0.34–0.78)^b^	2.0E-03	0.678
Prostate cancer	1.14 (0.56–2.30)	0.720	
Meta-analysis of all studies	0.67 (0.53–0.85)^b^	1.0E-03	0.438

^a^OR, odds ration; CI, confidence interval. *P* was for addictive model.

^b^OR and 95%CI were estimated from a fixed-effects model.

^c^*P* value of Cochran’s Q-test for the heterogeneity.

^d^NA, not available.

## References

[b1] Torre L. A. *et al.* Global cancer statistics, 2012. CA Cancer J Clin 65, 87–108 (2015). 25651787 10.3322/caac.21262

[b2] Rini B. I., Campbell S. C. & Escudier B. Renal cell carcinoma. Lancet 373, 1119–1132 (2009). 19269025 10.1016/S0140-6736(09)60229-4

[b3] Ferlay J. *et al.* Estimates of worldwide burden of cancer in 2008: GLOBOCAN 2008. Int J Cancer 127, 2893–2917 (2010). 21351269 10.1002/ijc.25516

[b4] Keizman D., Maimon N., Mishaeli M., Kuchuk I. & Gottfried M. [the Current Approach to Metastatic Renal Cell Carcinoma]. Harefuah 154, 535–539 (2015). 26480622

[b5] Melkonian S. C. *et al.* Gene-environment interaction of genome-wide association study-identified susceptibility loci and meat-cooking mutagens in the etiology of renal cell carcinoma. Cancer 122, 108–115 (2016). 26551148 10.1002/cncr.29543 PMC5016565

[b6] Mullins N. *et al.* Polygenic interactions with environmental adversity in the aetiology of major depressive disorder. Psychol Med 46, 759–770 (2016). 26526099 10.1017/S0033291715002172 PMC4754832

[b7] Melkonian S. C. *et al.* Joint association of genome-wide association study-identified susceptibility loci and dietary patterns in risk of renal cell carcinoma among non-Hispanic whites. Am J Epidemiol 180, 499–507 (2014). 25053674 10.1093/aje/kwu158 PMC4143080

[b8] Zawistowski M. *et al.* Extending rare-variant testing strategies: analysis of noncoding sequence and imputed genotypes. Am J Hum Genet 87, 604–617 (2010). 21070896 10.1016/j.ajhg.2010.10.012 PMC2978957

[b9] Mancikova V. *et al.* Thyroid cancer GWAS identifies 10q26.12 and 6q14.1 as novel susceptibility loci and reveals genetic heterogeneity among populations. Int J Cancer 137, 1870–1878 (2015). 25855579 10.1002/ijc.29557

[b10] Amin Al Olama A. *et al.* Multiple novel prostate cancer susceptibility signals identified by fine-mapping of known risk loci among Europeans. Hum Mol Genet 24, 5589–5602 (2015). 26025378 10.1093/hmg/ddv203 PMC4572072

[b11] Figueroa J. D. *et al.* Genome-wide association study identifies multiple loci associated with bladder cancer risk. Hum Mol Genet 23, 1387–1398 (2014). 24163127 10.1093/hmg/ddt519 PMC3919005

[b12] Rafnar T. *et al.* Sequence variants at the TERT-CLPTM1L locus associate with many cancer types. Nat Genet 41, 221–227 (2009). 19151717 10.1038/ng.296 PMC4525478

[b13] Purdue M. P. *et al.* Genome-wide association study of renal cell carcinoma identifies two susceptibility loci on 2p21 and 11q13.3. Nat Genet 43, 60–65 (2011). 21131975 10.1038/ng.723 PMC3049257

[b14] Chung C. C., Magalhaes W. C., Gonzalez-Bosquet J. & Chanock S. J. Genome-wide association studies in cancer–current and future directions. Carcinogenesis 31, 111–120 (2010). 19906782 10.1093/carcin/bgp273 PMC2860704

[b15] Onodera K. *et al.* Low-Frequency IL23R Coding Variant Associated with Crohn’s Disease Susceptibility in Japanese Subjects Identified by Personal Genomics Analysis. PLoS One 10, e0137801 (2015). 26375822 10.1371/journal.pone.0137801 PMC4574159

[b16] McCarthy M. I. *et al.* Genome-wide association studies for complex traits: consensus, uncertainty and challenges. Nat Rev Genet 9, 356–369 (2008). 18398418 10.1038/nrg2344

[b17] Wang Y. *et al.* Rare variants of large effect in BRCA2 and CHEK2 affect risk of lung cancer. Nat Genet 46, 736–741 (2014). 24880342 10.1038/ng.3002 PMC4074058

[b18] Davis L. & Maizels N. Homology-directed repair of DNA nicks via pathways distinct from canonical double-strand break repair. Proc Natl Acad Sci USA 111, E924–E932 (2014). 24556991 10.1073/pnas.1400236111 PMC3956201

[b19] Suwaki N., Klare K. & Tarsounas M. RAD51 paralogs: roles in DNA damage signalling, recombinational repair and tumorigenesis. Semin Cell Dev Biol 22, 898–905 (2011). 21821141 10.1016/j.semcdb.2011.07.019

[b20] Bakhoum S. F., Kabeche L., Murnane J. P., Zaki B. I. & Compton D. A. DNA-damage response during mitosis induces whole-chromosome missegregation. Cancer Discov 4, 1281–1289 (2014). 25107667 10.1158/2159-8290.CD-14-0403 PMC4221427

[b21] Delahaye-Sourdeix M. *et al.* A rare truncating BRCA2 variant and genetic susceptibility to upper aerodigestive tract cancer. J Natl Cancer Inst 107, doi: 10.1093/jnci/djv037 (2015). PMC4822523 25838448

[b22] Hoeijmakers J. H. Genome maintenance mechanisms for preventing cancer. Nature 411, 366–374 (2001). 11357144 10.1038/35077232

[b23] Bodmer W. & Bonilla C. Common and rare variants in multifactorial susceptibility to common diseases. Nat Genet 40, 695–701 (2008). 18509313 10.1038/ng.f.136 PMC2527050

[b24] Cybulski C. *et al.* CHEK2 is a multiorgan cancer susceptibility gene. Am J Hum Genet 75, 1131–1135 (2004). 15492928 10.1086/426403 PMC1182149

[b25] Cavanagh H. & Rogers K. M. The role of BRCA1 and BRCA2 mutations in prostate, pancreatic and stomach cancers. Hered Cancer Clin Pract 13, 16 (2015). 26236408 10.1186/s13053-015-0038-x PMC4521499

[b26] Yu Y. *et al.* Targeted DNA Sequencing Detects Mutations Related to Susceptibility among Familial Non-medullary Thyroid Cancer. Sci Rep 5, 16129 (2015). 26530882 10.1038/srep16129 PMC4632085

[b27] Lerner-Ellis J., Khalouei S., Sopik V. & Narod S. A. Genetic risk assessment and prevention: the role of genetic testing panels in breast cancer. Expert Rev Anticancer Ther 15, 1315–1326 (2015). 26523341 10.1586/14737140.2015.1090879

[b28] Liu C., Wang Q. S. & Wang Y. J. The CHEK2 I157T variant and colorectal cancer susceptibility: a systematic review and meta-analysis. Asian Pac J Cancer Prev 13, 2051–2055 (2012). 22901170 10.7314/apjcp.2012.13.5.2051

[b29] Martin S. T. *et al.* Increased prevalence of the BRCA2 polymorphic stop codon K3326X among individuals with familial pancreatic cancer. Oncogene 24, 3652–3656 (2005). 15806175 10.1038/sj.onc.1208411

[b30] Stern M. C. *et al.* Polymorphisms in DNA repair genes, smoking, and bladder cancer risk: findings from the international consortium of bladder cancer. Cancer Res 69, 6857–6864 (2009). 19706757 10.1158/0008-5472.CAN-09-1091 PMC2782435

[b31] Colin P. *et al.* Environmental factors involved in carcinogenesis of urothelial cell carcinomas of the upper urinary tract. BJU Int 104, 1436–1440 (2009). 19689473 10.1111/j.1464-410X.2009.08838.x

[b32] Hall J. *et al.* The associations of sequence variants in DNA-repair and cell-cycle genes with cancer risk: genotype-phenotype correlations. Biochem Soc Trans 37, 527–533 (2009). 19442246 10.1042/BST0370527

[b33] Lindahl T. & Wood R. D. Quality control by DNA repair. Science 286, 1897–1905 (1999). 10583946 10.1126/science.286.5446.1897

[b34] Brennan P. *et al.* Uncommon CHEK2 mis-sense variant and reduced risk of tobacco-related cancers: case control study. Hum Mol Genet 16, 1794–1801 (2007). 17517688 10.1093/hmg/ddm127

[b35] Yang S., Kuo C., Bisi J. E. & Kim M. K. PML-dependent apoptosis after DNA damage is regulated by the checkpoint kinase hCds1/Chk2. Nat Cell Biol 4, 865–870 (2002). 12402044 10.1038/ncb869

[b36] Falck J. *et al.* Functional impact of concomitant versus alternative defects in the Chk2-p53 tumour suppressor pathway. Oncogene 20, 5503–5510 (2001). 11571648 10.1038/sj.onc.1204811

[b37] Falck J., Mailand N., Syljuasen R. G., Bartek J. & Lukas J. The ATM-Chk2-Cdc25A checkpoint pathway guards against radioresistant DNA synthesis. Nature 410, 842–847 (2001). 11298456 10.1038/35071124

[b38] Hirotsu Y. *et al.* Multigene panel analysis identified germline mutations of DNA repair genes in breast and ovarian cancer. Mol Genet Genomic Med 3, 459–466 (2015). 26436112 10.1002/mgg3.157 PMC4585454

[b39] Chalermrujinanant C., Michowski W., Sittithumcharee G., Esashi F. & Jirawatnotai S. Cyclin D1 promotes BRCA2-Rad51 interaction by restricting cyclin A/B-dependent BRCA2 phosphorylation. Oncogene 35, 2815–2823 (2016). 26387543 10.1038/onc.2015.354 PMC6538526

[b40] Fridlich R., Annamalai D., Roy R., Bernheim G. & Powell S. N. BRCA1 and BRCA2 protect against oxidative DNA damage converted into double-strand breaks during DNA replication. DNA Repair (Amst) 30, 11–20 (2015). 25836596 10.1016/j.dnarep.2015.03.002 PMC4442488

[b41] Tookman L. A. *et al.* RAD51 and BRCA2 Enhance Oncolytic Adenovirus Type 5 Activity in Ovarian Cancer. Mol Cancer Res 14, 44–55 (2016). 26452665 10.1158/1541-7786.MCR-15-0188-T PMC4716290

[b42] Thompson E. R. *et al.* Reevaluation of the BRCA2 truncating allele c.9976A > T (p.Lys3326Ter) in a familial breast cancer context. Sci Rep 5, 14800 (2015). 26455428 10.1038/srep14800 PMC4601142

[b43] Esashi F., Galkin V. E., Yu X., Egelman E. H. & West S. C. Stabilization of RAD51 nucleoprotein filaments by the C-terminal region of BRCA2. Nat Struct Mol Biol 14, 468–474 (2007). 17515904 10.1038/nsmb1245

[b44] Kaczmarek-Rys M. *et al.* The c.470 T > C CHEK2 missense variant increases the risk of differentiated thyroid carcinoma in the Great Poland population. Hered Cancer Clin Pract 13, 8 (2015). 25798211 10.1186/s13053-015-0030-5 PMC4367841

[b45] Wojcicka A. *et al.* Variants in the ATM-CHEK2-BRCA1 axis determine genetic predisposition and clinical presentation of papillary thyroid carcinoma. Genes Chromosomes Cancer 53, 516–523 (2014). 24599715 10.1002/gcc.22162 PMC4058861

[b46] Delaneau O., Zagury J. F. & Marchini J. Improved whole-chromosome phasing for disease and population genetic studies. Nat Methods 10, 5–6 (2013). 23269371 10.1038/nmeth.2307

[b47] Howie B. N., Donnelly P. & Marchini J. A flexible and accurate genotype imputation method for the next generation of genome-wide association studies. PLoS Genet 5, e1000529 (2009). 19543373 10.1371/journal.pgen.1000529 PMC2689936

[b48] Marchini J., Howie B., Myers S., McVean G. & Donnelly P. A new multipoint method for genome-wide association studies by imputation of genotypes. Nat Genet 39, 906–913 (2007). 17572673 10.1038/ng2088

[b49] Rothman N. *et al.* A multi-stage genome-wide association study of bladder cancer identifies multiple susceptibility loci. Nat Genet 42, 978–984 (2010). 20972438 10.1038/ng.687 PMC3049891

[b50] Yeager M. *et al.* Genome-wide association study of prostate cancer identifies a second risk locus at 8q24. Nat Genet 39, 645–649 (2007). 17401363 10.1038/ng2022

