# Supplementary material for: Rare variants in BRCA2 and CHEK2 are associated with the risk of urinary tract cancers
Source: Sci Rep. 2016 Sep 16;6:33542. doi: 10.1038/srep33542 (PMC5025839; doi:10.1038/srep33542)

**Rare variants in *BRCA2* and *CHEK2* are associated with the risk of** **urinary tract cancers**

Yuqiu Ge^1,2^, Yunyan Wang^3^, Wei Shao^1,2^, Jing Jin^1,2^, Mulong Du^1,2^, Gaoxiang Ma^1,2^, Haiyan Chu^1,2^, Meilin Wang^1,2^,*, Zhengdong Zhang^1,2^,*

^1^Department of Environmental Genomics, Jiangsu Key Lab of Cancer Biomarkers, Prevention and Treatment, Collaborative Innovation Center For Cancer Personalized Medicine,Nanjing Medical University, Nanjing, China

^2^Department of Genetic Toxicology, The Key Laboratory of Modern Toxicology of Ministry of Education, School of Public Health, Nanjing Medical University, Nanjing, China

^3^Department of Urology, Huai-An First People’s Hospital Afﬁliated to Nanjing Medical University, Huai-An, China

Yuqiu Ge, Yunyan Wang, Wei Shao should be regarded as joint ﬁrst authors.

***Correspondence to:** Zhengdong Zhang, Department of Environmental Genomics, School of Public Health, Nanjing Medical University, 101 Longmian Road, Jiangning District, Nanjing 211166, China; Fax:+86-25-86868499; Email: drzdzhang@gmail.com, or Meilin Wang, Email: mwang@njmu.edu.cn

**Supplementary Figure legends**

**Supplementary Figure 1** Principal component analysis (PCA) plot for the study cohorts. **(A), (B)** and **(C)** show the principal component in bladder, renal and prostate cancer studies. **(D)** shows the principal components derived from three urinary tract cancers cohorts. The reference populations from 1000 Genomes Project samples are also included. X-axis is principal component 1 and y-axis is principal component 2. Cases and controls are clustering together.

**Supplementary Figure 1**


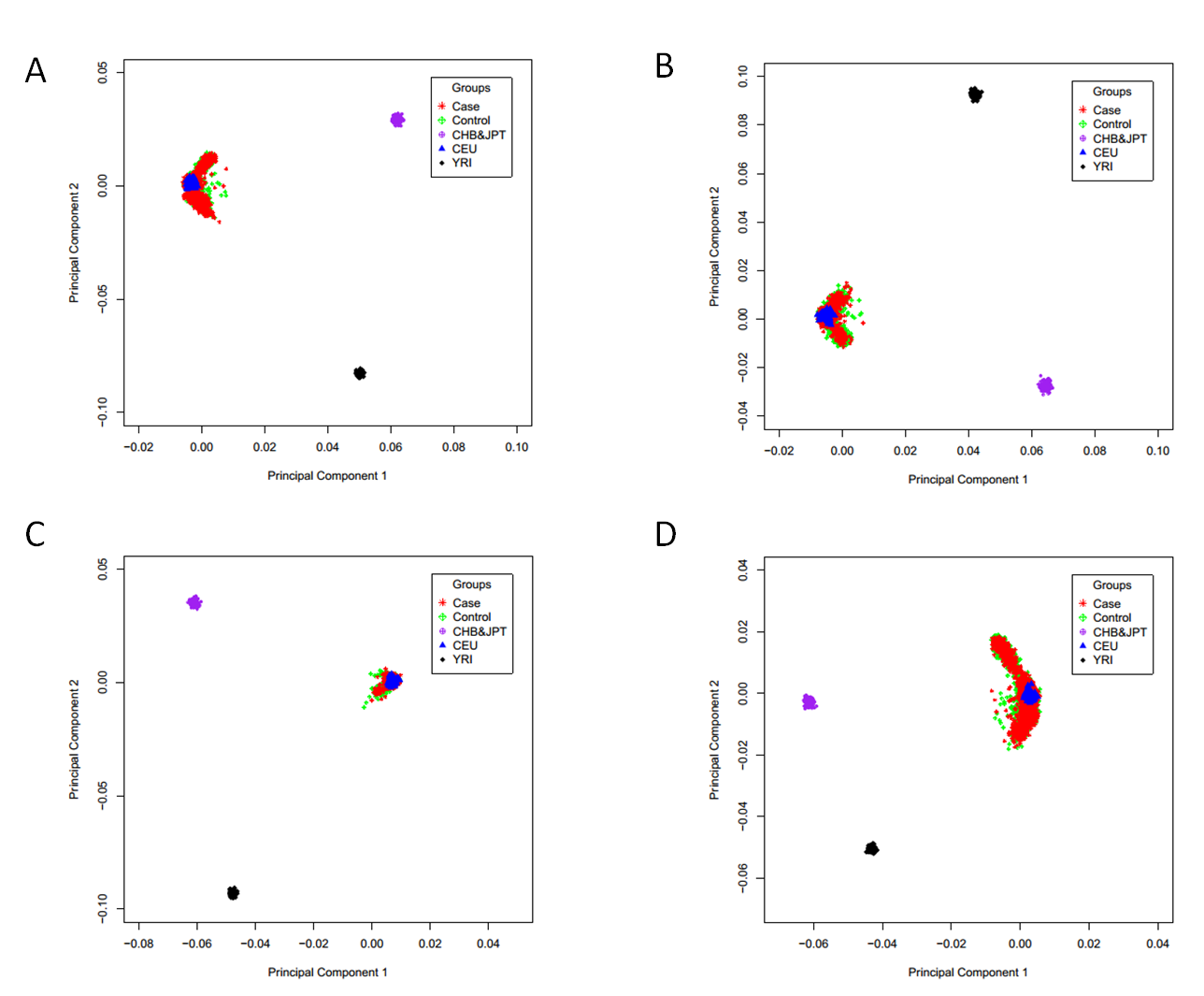

Supplement: Supplementary Information [file srep33542-s1.doc]
